# Persistent Lateral Knee Pain From a 10% Thickness Anterior Cruciate Ligament (ACL) Tear in a 36-Year-Old Runner

**DOI:** 10.7759/cureus.73081

**Published:** 2024-11-05

**Authors:** Edith (Edie) Sperling

**Affiliations:** 1 Anatomical Sciences, Western University of Health Sciences, Lebanon, USA

**Keywords:** anterior cruciate ligament (acl), endurance athlete, female athlete, overuse injury, partial-thickness tear, persistent knee pain, young adult female

## Abstract

Anterior cruciate ligament (ACL) tears are due to jumping, rapid decelerating, or quick changes in direction, but recent research indicates that accumulated tissue fatigue from repetitive submaximal knee loading can also cause ACL disruption. Partial degenerative ACL tears due to overuse are currently thought to be asymptomatic until the ligament is at least 50% torn. In this case, a 36-year-old female runner presenting with sharp lateral joint line knee pain, which precluded running or hiking over one mile, was found to have an atraumatic 10% thickness ACL tear. After ACL debridement, the patient was able to return to full activity. Females may be more prone to degenerative partial ACL tears due to biomechanical and hormonal factors. Partial ACL tears should be considered in the differential diagnosis of knee pain even without a history of trauma.

## Introduction

Isolated anterior cruciate ligament (ACL) injuries of the knee are common, with an estimated incidence of 200,000 per year in the US, of which half receive arthroscopic reconstruction [1]. Nearly three-quarters of ACL injuries involve minimal or no contact [2]. Partial ACL tears account for approximately 5-27% of ACL tears and are often missed by clinical orthopedic tests because of the presence of a firm end-feel [1]. Magnetic resonance imaging (MRI) may assist with diagnosis but can miss minor tears, leaving direct evaluation of the ACL during arthroscopic surgery to provide a definitive diagnosis [1,3].

Female athletes sustain noncontact ACL injuries between two and eight times the rate of male athletes, which has been hypothesized to be due to a greater Q angle (angle from the anterior superior iliac spine to the midline of the patella), general joint laxity, and the hormonal effects of estrogen [2,4]. While characteristic motions like a twist on a planted foot, a swift change in direction (cutting), and landing from a jump are known to increase the risk of an ACL tear, recent in vitro studies show that ACL failure can also be caused by accumulated tissue fatigue from repetitive submaximal knee loading [5-7].

Typical symptoms of an acute, isolated ACL disruption are well-known and include general knee pain, effusion, and loss of hyperextension and flexion, but symptoms of a partial, atraumatic ACL tear are not as well documented [5]. Partial ACL tears are generally thought to be symptomatic only if the ligament has at least a 50% thickness tear [8]. This case presents a 36-year-old female runner with sharp right lateral joint line knee pain of one-month duration. The aim of the case report is to highlight the possibility of lateral knee pain originating from partial, atraumatic ACL tears of less than 50% thickness.

## Case presentation

A 36-year-old female runner presented to the sports medicine clinic with a one-month history of right lateral knee pain when attempting to run or hike longer than one mile. The pain was described as sharp and localized to the lateral joint line. The onset was insidious. The patient denied a history of trauma and was otherwise healthy. She reported a typical mileage of 15-20 miles per week, primarily on trails. No edema was noted, and the ballottement was negative. Range of motion was within normal limits and without pain. Orthopedic knee tests were negative, including Lachman, anterior drawer, posterior drawer, pivot shift, Thessaly, McMurray, Apley’s grind, and Noble test [9]. Ober’s test was positive bilaterally for shortened iliotibial bands and the Thomas test was positive bilaterally for shortened iliopsoas and rectus femoris muscles [10,11]. The patient denied tenderness to palpation at knee structures including patellar tendon, lateral collateral ligament (LCL), medial collateral ligament (MCL), Gerdy’s tubercle, pes anserine, and joint line [12]. X-rays were unremarkable.

A diagnostic intraarticular lidocaine injection was given, and the patient was instructed to run to the point of typical symptom onset (over one mile) to determine if the issue was intraarticular [13]. The lidocaine injection was successful in temporarily eliminating the pain and thus was followed with an intraarticular cortisone injection. The cortisone injection was only partially effective for less than one week. Thereafter, the patient subsequently completed six weeks of outpatient orthopedic physical therapy, twice a week. The physical therapy evaluation included functional biomechanical assessment, running gait assessment, strength testing, and joint mobility testing. The patient was found to have a mild Trendelenburg sign (left hip drop with right single-leg stance) and mild left genu valgus with dynamic activity such as running and single-leg hops; both the Trendelenburg and genu valgus became more pronounced with fatigue [13].

**Figure 1:**
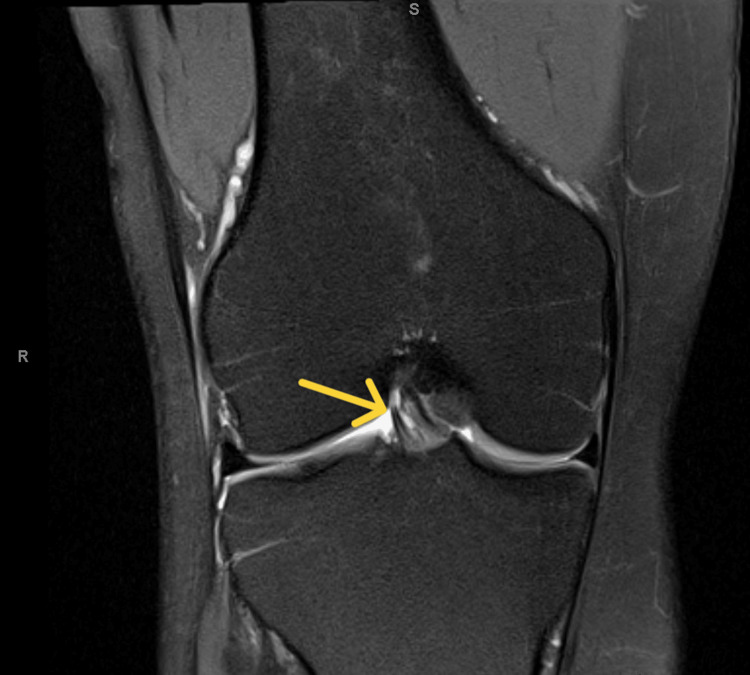
AP MRI of ACL exhibiting 10% tear AP, anterioposterior; ACL, anterior cruciate ligament; MRI, magnetic resonance imaging

**Figure 2:**
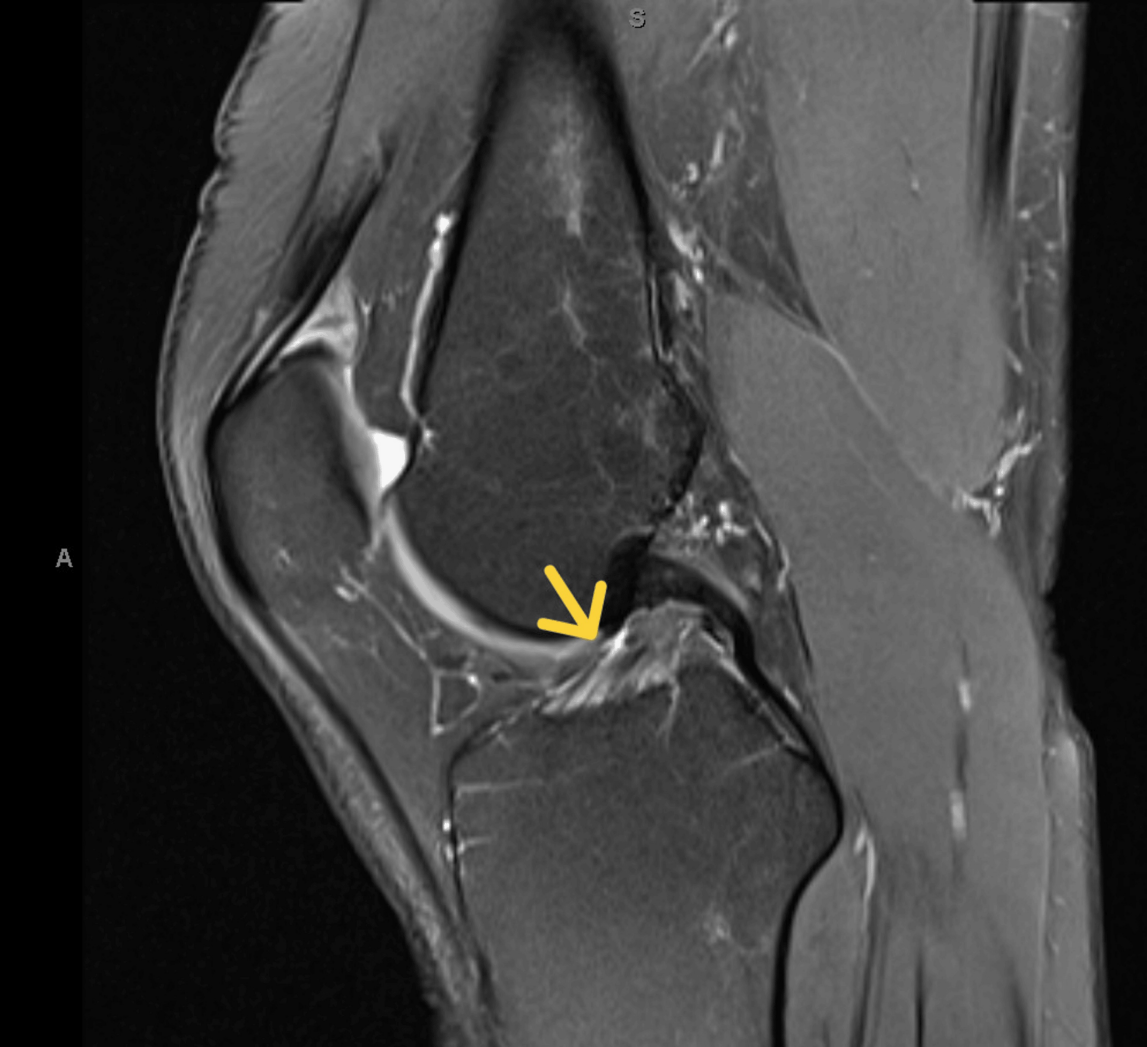
Lateral MRI of ACL exhibiting 10% tear ACL, anterior cruciate ligament; MRI, magnetic resonance imaging

The patient was treated with neuromuscular re-education to address hip drop and genu valgus, strengthening of the lower extremity musculature with a focus on the gluteus medius to reduce Trendelenburg gait, manual therapy for moderate restriction in right ankle dorsiflexion, and general cardiovascular conditioning to prevent fitness loss [13]. Physical therapy was also unsuccessful in changing or reducing the lateral knee pain. The patient was consequently referred to an orthopedic surgeon and an MRI was ordered, which demonstrated a 10% thickness tear of the right ACL (Figures 1, 2). The ACL tear was thought to be too minor to be the cause of the pain, but an exploratory arthroscopy was performed of the right knee with the expectation that another origin would present a diagnosis [14]. During surgery, the knee was found to be globally intact, with the 10% thickness ACL tear being the only pathology. The tear was debrided, which resulted in the elimination of symptoms and a timely return to full activity without pain.

## Discussion

This case study demonstrates the resolution of lateral joint line knee pain following arthroscopic debridement of a 10% thickness, atraumatic ACL tear, which was likely due to accumulated tissue fatigue from repetitive submaximal knee loading, i.e., overuse [7]. Arthroscopy was undertaken after the failure of an intraarticular cortisone injection and six weeks of physical therapy to resolve symptoms. After two weeks of non-weight-bearing, followed by four weeks of 50% weight-bearing and four weeks of physical therapy for strengthening and biomechanical training, the patient gradually returned to full-distance running and hiking without pain, indicating that the small ACL tear was the cause of the symptoms. It is likely that the patient’s mild right gluteus medius weakness contributed to genu valgus in the right knee, which, over time and with increased mileage, caused enough cumulative stress on the ACL to result in a partial tear. Genu valgus is known to put stress on the ACL [4].

Full ACL ruptures often result in knee instability and dysfunction [2-5]. Individuals who are able to strengthen the surrounding musculature sufficiently to regain neuromuscular control and joint stability are referred to as “copers,” i.e., they cope well without surgery, while those who fail to regain stability and elect to undergo surgical repair are referred to as “noncopers” [15]. It may be that small partial ACL tears also result in copers and noncopers, whereby some individuals are asymptomatic or are able to regain full stability, and some are not. Physical therapy can be very useful in addressing biomechanical issues and neuromuscular retraining to treat knee pain; however, a small partial ACL tear may require surgical debridement in some cases [16].

ACL disruption from overuse may be more common in females than males, as female ACLs are typically at a mechanical disadvantage due to greater Q angle and higher overall joint laxity [2]. Literature describing partial, atraumatic ACL tears is lacking, so it is not known if female and male anatomy present differing incidence, prevalence, mechanism of injury, prognosis, or recovery trajectory, but it is probable that partial atraumatic ACL tears follow the same pattern as full-thickness ACL tears and females are more at risk.

## Conclusions

While it is generally thought that partial ACL tears need at least a 50% thickness tear to be symptomatic, this patient case demonstrates that a small, atraumatic ACL disruption can cause persistent lateral joint line knee pain. ACL disruption from overuse may be more common in females than males, as females are more at risk for full-thickness ACL disruption due to a variety of factors. However, research on partial ACL tears due to overuse is scarce, and further research is warranted. Healthcare providers may benefit from incorporating atraumatic partial ACL tear in their differential for lateral knee pain.
